# After the Octopus; Not Entitled to Second-class Mail Rates

**Published:** 1906-05

**Authors:** 


After the Octopus; Not Entitled to Second-class Mail Rates.—In reply to a specific inquiry, the Third Assistant Postmaster General writes me: "If the *Journal of the American Medical Association* is, as stated in the clipping sent by you, sent free to members of the American Medical Association, it would appear that such persons are not actual subscribers."

[This refers to Circular No. 25, from office of Third Assistant Postmaster General, December 16, 1905, stating that to be entitled to pound rates every publication must have a bona fide paid subscription list, and that sending free copies at pound rates to members of an association is a violation of the postal laws. See April "Red Back."]

---

Said the little Red Rooster to the little Red Hen,
"You haven't laid an egg in I don't know when."
Said the little Red Hen to the little Red Rooster,
"You don't come around as often as you used to."

